# Often neglected steps in transforming drug solubility from single measurement in pure water to physiologically-appropriate solubility-pH

**DOI:** 10.5599/admet.2626

**Published:** 2025-02-26

**Authors:** Alex Avdeef

**Affiliations:** in-ADME Research, New York City, NY 10128, USA

**Keywords:** Constant ionic medium, weakly-ionizable drugs, calculated saturation pH, Stokes-Robinson hydration theory, salting-out, ambient dissolved CO_2_, analytic continuation to a full solubility-pH profile

## Abstract

**Background and purpose:**

The solubility of weakly-ionizable drugs in pure water, S_w_, is commonly measured. The pH-dependent properties of the saturated solutions can be surprisingly complex in subtle ways. This commentary examines the characteristics of such measurements through case studies of 32 free acids, bases, and ampholytes (including crocetin, glibenclamide, mellitic acid, quercetin, bedaquiline, brigatinib, imatinib, celecoxib, and lysine), using published water solubility data.

**Computational approach:**

Usually, in such saturated solutions, the ionic strength, *I*_w_, is close to zero. When the pH is adjusted away from pH_w_, the ionic strength increases, substantially in some cases (*e.g. I*_w_ > 10 M at pH 7.4 for mellitic acid and lysine). This change in ionic strength alters the activities of the species in solution. The corresponding equilibrium constants used to calculate the concentrations of these species must be adjusted accordingly. Here, the Stokes-Robinson hydration theory, slightly modified with Setschenow ‘salting-out’ constants to account for solvent interactions with unionized drugs, was used to estimate activity coefficients. The calculations were performed with the pDISOL-X program.

**Key results:**

Given reliably-measured values of solubility in water (*S*_w_) and ionization constant (p*K*_a_) of the drugs and assuming that the Henderson-Hasselbalch equation is valid, a method is described for (i) adjusting the measured *S*_w_ values at ionic strength, *I*_w_ ~ 0 M, to values expected at reference ionic strength, *I*_ref_ = 0.15 M (or at any other reasonable reference value), (ii) determining the water pH_w_ in saturated solutions of added neutral-form drugs; (iii) determining the intrinsic solubility, *S*_0_, both at *I*_w_ and *I*_ref_, and (iv) using analytic-continuation in the equilibrium mass action model to deduce the solubility values as a function of pH, harmonized to a selected *I*_ref_. For highly soluble drugs, whose *I*_w_ exceeds 0.15 M, the intrinsic solubility values appear to depend on the amount of excess solid added.

**Conclusion:**

This commentary re-emphasizes that measured *S*_w_ is not generally the same as *S*_0_. It is stressed that transforming measured drug solubility in pure water to an ionic strength level that is physiologically appropriate would better match the conditions found in biological media, potentially improving applications of solubility in pharmaceutical research and development.

## Introduction

In drug discovery, the solubility of research compounds in pure water is a necessary initial measurement for predicting how well a test compound may dissolve in aqueous media and thus be available for absorption. In cases of newly approved drugs, access to the measured data is limited. Finding reliable solubility data can be a challenge: frequently the only sources of solubility information are found in patents and regulatory agency public reports [1-3]. The experimental conditions are sparsely detailed in such citations. The measurement temperature is not always stated. The pH of the saturated solution, pH_w_, is hardly ever reported. Too frequently, sufficient procedural details are not revealed even in some journal publications. There would appear to be many opportunities for elevating the ‘state of the art.’

As part of an ongoing interest in enhancing the quality of solubility measurements of weakly-ionizable druglike molecules [4-7], this commentary addresses the measurements of *unionized* drug solubility in pure water, *S*_w_, and how such measurements can be realistically adjusted to physiologically-relevant pH range and ionic strength. This is a focused discussion: only equilibrium solubility of neutral-form solids is addressed. Drug salts are not considered here; it is assumed that complications such as aggregation or adsorption to solid surfaces do not occur to any significant extent (*i.e.* the simple Henderson-Hasselbalch equation can be assumed to be valid).

In ideal practice [4-7], using the phase-solubility method [8], a pure weakly-ionizable drug (acid, base, or ampholyte) in neutral form is added to freshly-distilled/purified water (CO_2_-free and without buffer) to the extent that a stable saturated suspension is achieved, for a reported precise quantity of drug added (noting that some equilibrium reactions can depend on the total amount of excess solid added). The suspension, thermostated to a selected temperature (usually 25 or 37 °C), is allowed to fully equilibrate, which may take 24 h (or longer in the case of practically insoluble drugs). After equilibration is reached, the saturation pH (pH_w_) is precisely measured using a research-grade electrode calibrated and standardized for *research* purposes [9]. Before the concentration of the sparingly-soluble drug dissolved in water is measured, the solid-liquid separation procedure needs to ensure that the concentration of the drug in the aqueous phase is not significantly lowered by adsorption to surfaces of vials/filters in the separation apparatus [5].

When a neutral weakly ionizable drug is added to freshly distilled water to form a saturated solution, the pH of the suspension can be substantially altered from the water pH ~7, depending on the p*K*_a_ and the intrinsic solubility of the drug. For sparingly soluble molecules, the measured solubility, *S*_w_, can be expected to far *exceed* that of the intrinsic (neutral form) solubility, *S*_0_ [10]. For multiprotic molecules with overlapping p*K*_a_ values (*e.g.* quercetin, mellophanic, and mellitic acids), the relationships between *S*_w_ and S_0_ can be complicated.

Even in ideal practices, there are still neglected considerations. These can potentially lead to misinterpretations and misapplications of *S*_w_, as will be addressed in this commentary. For example, the ionic strength of the saturated solutions, *I*_w_, in the measurements is practically zero, with some exceptions. In contrast, the physiologically-relevant ionic strength, *I*_ref_, is often taken to be 0.15 M. In quantitative applications of *S*_w_ to bio-relevant systems, this gap between *I*_w_ and *I*_ref_ needs to be factored in.

The relationships between S_w_, *S*_0_, pH_w_, and p*K*_a_ are rigorously explored here. The intrinsic solubility, *S*_0_, is indicated at the pH where a relatively soluble molecule is practically at zero net charge. If the p*K*_a_ and *S*_w_ are known and the Henderson-Hasselbalch (HH) equation is valid in the case, it is possible to calculate the saturation pH_w_, as well as *S*_0_. The details of such calculations and supportive topics are presented in Appendices A-C. For the simplest cases (mono- and diprotic ionizable molecules), examples of derivations of explicit equations are presented in Appendix C, with examples given below. These equations best serve as checks on the general procedures developed here. All mass action calculations here were performed using general nonlinear regression techniques, based on implicit equations, derived internally using *p*DISOL-X.

Abraham and Le [10] discussed the relationship between the measured *S*_w_ and *S*_0_ and how the difference between them depends on the measured pH_w_. For a weak acid, the HH equation (Eq. A9 in Appendix C) is *S*_w_ = *S*_0_ (1 + 10^-p*K*a+pHw^). It may be approximated (cf., Eq. A13) that pH_w_ ≈ ½ (p*S*_0_ + p*K*_a_). For a very soluble weak acid, such as acetaminophen, a further simplification yields pH_w_ ≈ ½p*K*_a_. The corresponding HH equation reduces to *S*_w_ ≈ *S*_0_(1 + *K*_a_^½^), where to a good approximation, *S*_w_ ≈ *S*_0_ (*cf.* Tables 1, 2). In contrast, for a practically insoluble weak acid (*e.g.* crocetin), the predicted approximate pH_w_ depends on both p*S*_0_ and p*K*_a_. Substitution of pH_w_ ≈ ½ (p*S*_0_ + p*K*_a_) into the HH equation indicates that *S*_w_ is about 20 time greater than *S*_0_ in the case of *crocetin* (*cf.* Tables 1, 2).

**Table 1.** p*K*_a_ of acids, bases, and ampholytes at ionic strength *I*_ref_ = 0.15 M and *I*_w_ in saturated water solutions at pH_w_^a^

Substance	Type^b^	*t* / °C	*I*_w_ / mM^c^		p*K*_a1_^ref^	p*K*_a1_^*I*^		p*K*_a2_^ref^	p*K*_a2_^*I*^		p*K*_a3_^ref^	p*K*_a3_^*I*^		p*K*_a4_^ref^	p*K*_a4_^*I*^		p*K*_a5_^ref^	p*K*_a5_^*I*^		p*K*_a6_^ref^	p*K*_a6_^*I*^
Acetaminophen	A	37	0.010		**9.41**	9.62															
Crocetin	A	23	0.004		**4.47**	4.71		5.00	5.35												
Glibenclamide	A	37	0.017		**5.18**	5.40															
Indomethacin	A	37	0.046		**4.02**	4.24															
Isotretinoin	A	25	0.011		**4.52**	4.76															
Naproxen	A	37	0.112		**4.19**	4.40															
Phenytoin	A	37	0.005		**8.14**	8.36															
Quercetin	A	23	0.004		**7.12**	7.31		8.43	8.80		9.67	####		10.63	####						
Benzoic acid	A	25	1.4		**3.99**	4.17															
Phthalic acid, 2-	A	25	7.6		**2.71**	2.85		4.90	5.14												
Trimellitic acid	A	25	19		**2.34**	2.43		3.71	3.89		5.10	5.39									
Hemimellitic acid	A	25	23		**2.60**	2.69		3.78	3.95		5.45	5.71									
Pyromellitic acid	A	25	20		**1.86**	1.94		**2.74**	2.90		**4.28**	4.54		**5.33**	5.69						
Mellophanic acid	A	25	102		**2.00**	2.01		**3.11**	3.15		**4.51**	4.57		**5.91**	5.99						
Benzenepentacarboxylic acid	A	25	128		**1.74**	1.75		**2.60**	2.61		**3.76**	3.78		**4.97**	5.00		**6.10**	6.14			
Mellitic acid^d^	A	25	381		**1.10**	1.09		**1.69**	1.63		**2.75**	2.64		**4.00**	3.85		**5.05**	4.83		**6.04**	5.77
Mellitic acid^e^	A	25	486		**1.10**	1.09		**1.69**	1.62		**2.75**	2.62		**4.00**	3.80		**5.05**	4.77		**6.04**	5.69
Atenolol	B	37	1.7		**9.19**	9.14															
Bedaquiline	B	23	0.002		**8.77**	8.68															
Brigatinib	B	23	0.123		**1.73**	1.27		**3.65**	3.35		**4.72**	4.57		**8.04**	7.95						
Carvedilol	B	37	0.009		**7.78**	7.72															
Emtricitibine	B	25	0.001		**2.67**	2.63															
Imatinib	B	23	0.004		**1.71**	1.19		**3.10**	2.76		**3.88**	3.71		**8.03**	7.96						
Imiquimod	B	25	0.001		**3.55**	3.33		**6.54**	6.48												
Ketoconazole	B	37	0.001		**3.32**	3.16		**6.17**	6.09												
Lamotrigine	B	37	0.002		**5.24**	5.19															
Lumefanatrine	B	23	0.004		**9.35**	9.25															
Celecoxib	X	37	0.0002		**2.11**	2.05		**9.37**	9.59												
Enrofloxacin	X	37	0.086		**6.17**	6.11		**7.70**	7.91												
Lysine, L-^f^	X	27	695		**2.20**	2.36		**9.12**	9.24		**10.64**	####									
Lysine, L-^g^	X	27	612		**2.20**	2.34		**9.12**	9.22		**10.64**	####									
Mycophenolate Mofetil	X	37	0.038		**5.64**	5.56		**8.26**	8.50												
Piroxicam	X	37	0.019		**1.84**	1.80		**5.13**	5.33												
Sulfamethoxazole	X	37	0.024		**1.97**	1.92		**5.65**	5.86												

^a^p*K*_a_^ref^ refer to published values, harmonized to *I*_ref_ = 0.15 M; pK_a_^*I*^ are values transformed to *I*_w_

^b^ A = acid, B = base, X = ampholyte.

^c^*I* in neutral-drug saturated water

^d^Total added 9.5 g / mL (10-fold excess over solubility value)

^e^Total added 1.9 g / mL (2-fold excess)

^f^Total added 1.2 g / mL (2-fold excess over solubility value)

^g^Total added 0.6 g / mL (1.1-fold excess).

**Table 2.** Analysis results of solubility of ionizable acids in water (*I*_ref_ = 150 mM)

Weak acids	log (*S_w_*^Lit^ / M)	*S_w_*^Lit^ / mg mL^-1^	*t* / °C	Ref.	*K*_salt_^a^	pH_w_	log *(S_0_*^ref^/ M)	log (*S*_0_^*I*^ / M)	*I* / mM
Crocetin	-5.74	0.0006	23	[18]	0.38	5.56	-7.04	-6.99	0.004
Isotretinoin	-4.77	0.005	25	[19]	0.46	5.02	-5.26	-5.19	0.011
Quercetin	-4.07	0.026	23	[20]	0.11	6.07	-4.11	-4.09	0.004
Indomethacin	-4.14	0.026	37	[17]	0.34	4.39	-4.55	-4.50	0.046
Glibenclamide	-4.18	0.033	37	[17]	0.41	4.81	-4.33	-4.26	0.017
Phenytoin	-3.65	0.056	37	[17]	0.25	5.14	-3.69	-3.66	0.005
Naproxen	-3.38	0.097	37	[17]	0.27	4.00	-3.55	-3.51	0.112
Acetaminophen	-0.85	21	37	[17]	0.12	5.06	-0.87	-0.85	0.010
Benzoic acid	-1.55	3.5	25	[16]	0.15	2.92	-1.59	-1.57	1.4
2-Phthalic acid	-1.36	7.3	25	[16]	0.11	2.20	-1.46	-1.44	7.6
Trimellitic acid^b^	-1.00	21	25	[16]	0.08	1.82	-1.10	-1.09	19
Hemimellitic acid^b^	-0.62	51	25	[16]	0.08	1.73	-0.67	-0.66	23
Pyromellitic acid^c^	-1.37	11	25	[16]	0.04	1.79	-1.61	-1.61	20
Mellophanic acid^c^	0.02	264	25	[16]	0.04	1.14	-0.02	-0.02	102
Benzenepentacarboxylic acid	-0.05	271	25	[16]	0.00	1.06	-0.11	-0.11	128
Mellitic Acid^d^ (10-fold excess)	0.45	988	25	[16]	-0.04	0.40	0.32	0.33	381
Mellitic Acid^d^ (2-fold excess)						0.36	0.36	0.37	485

^a^Calculated using Eq. (A5b)

^b^Tricarboxyilic acid

^c^Tetracarboxylic acid

^d^Hexacarboxylic acid.

If measured compounds contain protogenic impurities (*e.g.* ambient CO_2_), the measured pH_w_ value may be affected substantially when the weakly ionizing drug has low self-buffering capacity. For example, when the aqueous solubility of a practically insoluble free base (p*K*_a_ > 9) is measured in water, the expected measured pH would be ~7 < pH_w_ < p*K*_a_ and barely affected by the tiny amount of the base dissolved. On the other hand, the measured pH can be substantially affected by CO_2_ dissolved in water. This effect is frequently not recognized (or sometimes ignored), especially if pH_w_ is not measured. For example, log *S*_w_ = -4.87 has been reported for terfenadine (p*K*_a_ 9.77) dissolved in water, with no further information [11]. In the terfenadine suspension, if the typical ambient [CO_2_] dissolved in water were 0, 10, or 20 μM, the calculated log *S*_0_ would be -5.7, -6.3, or -8.3, and the measured pH_w_ would be 9.2, 8.5, or 6.6, respectively in the three cases. Generally, the error in the calculated S_0_ of practically insoluble *basic* drugs in water is expected to be substantial (up to 2-3 log units), since it is very difficult to eliminate CO_2_ entirely simply by spurging the solution with an inert gas. Hence, with poorly-soluble basic drugs, it is critically important to measure the equilibrium saturation pH_w_. It is expected that experienced solution chemists recognize this possible source of error and thus routinely measure and report the equilibrium pH_w_ when *S*_w_ is determined.

For reliably-measured pH_w_, it is possible to estimate the ambient concentration of CO_2_ and correct for its effect. But, measuring the pH accurately in an unbuffered solution is yet another challenge, because a pH electrode, being an electrochemical device, requires a certain amount of electrical current for its operation, to stabilized potentially erratic reference electrode junction voltages. Electrodes in such applications need to be calibrated/standardized in special ways [12] (pp. 55-66, 130-145). Solution chemists can overcome this low conductivity challenge by adding a small amount of inert electrolyte to the suspension (*e.g.* 1-5 mM NaCl), which sufficiently lessens the electrode junction errors.

In this commentary, case studies of sixteen weak acids, ten weak bases, and six ampholytes are used to explore the solution mechanistic aspects of the subtle complexity derived from reported weakly-ionizable drug solubility measurements in pure water. Although less evident in low-soluble drugs, the ionic strength of a saturated solution of a strongly self-buffered soluble molecule can reach high values when pH is adjusted away from pH_w_ (*e.g. I* > 10 M at pH 7.4 for lysine and mellitic acid). This increase in *I* is due to increased ionization of the initially minimally-charged drug. The activities of all species involved change substantially, for which corrections can (and should) be made. The Stokes-Robinson hydration theory [13,14], slightly modified with Setschenow ‘salting-out’ constants [15, and references therein] to account for uncharged drug interactions with the solvent, was used to estimate activity coefficients. Given reliably-measured values of *S*_w_ and p*K*_a_ of a druglike molecule, a rigorous method is described here for (i) adjusting *S*_w_ from zero ionic strength to 0.15 M (or a similar practical value), (ii) calculating the saturation pH_w_, (iii) calculating the intrinsic solubility, *S*_0_, and (iv) using analytic-continuation in the equilibrium mass action model to deduce the solubility values as a function of pH at a selected reference ionic strength.

## Computational methods

### Literature data used

To probe the characteristics of solubility of neutral-form drugs added in excess to pure water, the measured S_w_ values of thirty-two weakly-ionizable druglike molecules were taken from published sources [16-27]. A systematic series of benzene carboxylic acids (benzoic, phthalic, trimellitic, hemimellitic, pyromellitic, mellophanic, benzene pentacarboxylic, and mellitic acid) and eight other weak *acids* covering a wide range of solubility values (acetaminophen, crocetin, glibenclamide, indomethacin, isotretinoin, naproxen, phenytoin, and quercetin) were selected. The ten selected weak *bases* include atenolol, bedaquiline, brigatinib, carvedilol, emtricitabine, imatinib, imiquimod, ketoconazole, lamotrigine, and lumefanatrine. The six *ampholytes* considered are celecoxib, enrofloxacin, L-lysine, mycophenolate mofetil, piroxicam, and sulfamethoxazole.

Values of p*K*_a_ were taken from standard compilations, matching the temperature of the solubility measurements and harmonizing on ionic strengths, as described below [28-34]. For newer drugs, relevant literature sources were consulted, including patents and public regulatory agency documents.

### Determination of equilibrium pH and intrinsic solubility

The solubility analysis, refinement, and simulation computer program, *p*DISOL-X™ (*in-ADME* Research), was used in this study. The mathematical approach based on a mass action equilibrium model continues to evolve [35-39]. The program has been effectively applied in several studies involving multifactorial equilibria: self-aggregation [40-45], cocrystals [46-48], complexation [49,50], and salt disproportionation [51,52]. Recently, salting-out activity corrections have been added [15].

The data analysis method uses log *S* (molarity basis) as measured input data, as a function of pH. The analytical concentrations of all added reagents are specified. The mass action algorithm considers the contribution of all species proposed to be present in the solution. The algorithm derives its own implicit polynomial equations internally, given a practical number of equilibrium reactions and the corresponding roughly estimated constants. The program refines the estimated constants and calculates the distribution of species and reactants consequent to a simulated sequence of additions of titrant (*e.g.* HCl or NaOH, or a few program-recognized ionizable titrants: phosphoric acid, maleic acid, lysine, *etc*.), to simulate the suspension pH speciation from pH 0 to 13. The ionic strength, *I*, is iteratively calculated at each pH. Values of p*K*_a_, intrinsic solubility, along with pH electrode standardization constants, are accordingly adjusted at each pH point for activity deviations from the benchmark level of *I*_ref_ = 0.15 M, selected as the basis of the ‘constant ionic medium’ thermodynamic state [12].

All the equilibrium constants reported here are based on the concentration scale, *i.e*. the ‘constant ionic medium’ thermodynamic standard state, without loss of thermodynamic rigor [12] (pp. 43-47). Since the measured pH is based on the ‘operational’ activity scale (p_a_H), such values need to be converted to the concentration scale, p_c_H (= -log *c*_H_+), as described elsewhere [12] (pp. 55-66; *cf.* Appendix B). In such a system, the electrode is first ‘calibrated’ using NIST standard pH buffers, which establishes the relationship between meter voltage (mV) readings and the p_a_H scale. These values are then converted into the p_c_H scale.

Since *I* at any given pH point in an acid-base titration is likely to be different from *I*_ref_, all ionization constants are locally transformed (from *I*_ref_ to local *I*) for the calculation of local point concentrations (as detailed in Supplementary material, Appendix A). It is a reasonable practice to designate 0.15 M as the benchmark ‘reference’ ionic strength, *I_ref_* (‘physiological’ level), to which all equilibrium constants are harmonized in the data analysis. The procedure uses activity corrections based on the hydration theory proposed by Stokes and Robinson [13,14,53-55], as described by examples in the next four sections and in further detail in Appendix A.

### Stokes-Robinson hydration theory modified to better account for the activity corrections of uncharged molecules

Activity corrections using the Stokes-Robinson hydration theory model (SRHT) [13] have seldom been applied in solution speciation studies involving multiprotic drugs in media containing numerous charged and neutral species. One exception appears to be that of Wang *et al.* [14].

We adapted the version detailed by Wang *et al.* into *p*DISOL-X in 2013 [40] and continue to make slight improvements to the treatment. In many of the classical papers embracing the SRHT method, most of the emphasis has been placed on correcting the activity of charged species, since long-range ion-ion interactions play a dominant role in solutions with *I* < 0.5 M, in comparison to the effect of short-range ion-solvent interactions. Activity corrections for neutral species are seldom discussed in the context of SRHT model. There are three components to the model (Eq. A4 in Appendix A). The first term in Eq. A4 addresses the dominant ion-ion electrostatic interactions. The impact of the SRHT model on uncharged species arises from the second and third ‘solvation’ terms in Eq. A4 (ion-solvent interactions), although the calculated activity coefficients are practically unit value in the model, unless *I* > 1 M.

As ions are added to pure water, some of the water molecules are removed from the initial pure water and taken up by the ions as part of their primary solvation shells. For example, hydrogen ions are thought to sequester seven water molecules into the solvation shell, but chloride ions only hold one water molecule as such [14]. More water is sequestered if the ions are multiply charged and arise from relatively soluble electrolytes (*e.g.* mellitic acid, lysine, *etc.*). So, the presence of charged species results in less free water to dissolve the neutral molecule, because some of the water is removed from the bulk solvent into the solvation shells of the ions. In terms of the total volume of water, the solubility of uncharged species appears to be reduced in the presence of a large concentration of solvated ions, since it is the free water volume that dictates the solubility equilibrium. The phenomenon is termed ‘salting out’ [15] (and references therein).

In our recent study of the impact of salting-out on equilibria involving self-association of an ionizable solute [15], a model to predict salting-out constants was introduced, as further described by Eq. A5 in Appendix A. In the present study, we compared the predicted activity coefficients of neutral molecules using the SRHT model (Eq. A4) with those arising from the salting-out equation (Eq. A5). In all the cases we considered, the legacy SRHT appears to underestimate the effect of ion-water interactions on the activity of uncharged species, in comparison to that of the salting-out effect.

In the present commentary, we propose a modified SRHT model, which incorporates predicted salting-out constants to estimate the activity coefficients of neutral molecules. The modified SRHT model is a tentative proposal, awaiting more direct experimental confirmation. Since salting-out studies have been performed far more often than applications of SRHT and since there are many measurements of the effect of salt on the solubility of uncharged species, our approach in this commentary may be an opportunity to improve the SRHT model for future applications involving druglike molecules.

### Example of the ‘constant ionic medium’ activity scale treatment in subsaturated solution

Consider the case of a monoprotic weak acid, acetaminophen, whose ionization constant at *I*_ref_ = 0.15 M and 37 °C is p*K*_a_^ref^ = 9.41. When 20 mg of pure acetaminophen are added to 1.0 mL of distilled water, the equilibrated pH = 5.29 and ionic strength *I* = 5.7 μM at the 132 mM subsaturation concentration. To perform mass action calculations in the near-zero ionic strength, the p*K*_a_^ref^ needs to be adjusted for the changes in activities between the two ionic strengths (5.7 μM and 0.15 M). The general Eq. A3 in Appendix A reduces to Eq. (1):



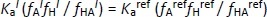

(1)


where *K_a_^I^* is the ionization constant at ionic strength, *I*; *f*_A_, *f*_H_, and *f*_HA_ are the activity coefficients of A^-^, H^+^, and HA. In the subsaturated solution, the activity coefficient of the neutral species, HA, is expected to be near unit value, independent of ionic strength. The activity coefficients of the charged species may be calculated using Eq. A4 (SRHT). Since both ionic strengths are relatively low, the first term of Eq. A4 (Debye-Hückel term) dominates. At *I* = 5.7 μM, the calculated activity coefficients are: *f*_A_^*I*^ = *f*_H_^*I*^ = 0.997, *f*_HA_^*I*^ ≡ 1. At *I*_ref_ = 0.15 M, the corresponding coefficients change to *f*_A_^ref^ = 0.769, *f*_H_^ref^ = 0.828, *f*_HA_^ref^ ≡ 1. Inserting these values into Eq. 1 produces Eqs (2a)



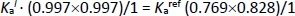

(2a)


In negative log form, Eq. (2b)



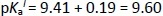

(2b)


### Example of the ‘constant ionic medium’ activity scale treatment in saturated solution

Next, consider the case of a *saturated* solution of the drug: when 25 mg of pure *acetaminophen* are added to 1.0 mL of distilled water, the calculated saturation pH 5.28 at the added *total* concentration of 164 mM, with *I* = 6.6 μM. The two equilibrium equations (in cumulative form, Eq. A2) are A + H (HA and A + H ( HA(s). The cumulative constant for the second equation, *β*_HA(s)_^ref^ = p*K*_a_^ref^ + p*S*_0_^ref^ = 9.41 +0.87 = 10.28.

The activity coefficient of the *solid* species, *f*_HA(s)_^*I*^ and *f*_HA(s)_^ref^ are generally defined as unit value. The salting-out constant of acetaminophen, *K*_salt_ = 0.117 M^-1^, is used to determine the activity of HA: log *f*_HA_^*I*^ = 0.117×6.66×0^-6^ = 7.72×10^-7^ or *f*_HA_^*I*^ = 1.000002; log *f*_HA_^ref^ = 0.117×0.15 = 0.0176 or *f*_HA_^ref^ = 1.04124. The activity coefficients of the ions are practically unchanged from the prior example: *f*_A_^*I*^ = *f*_H_^*I*^ = 0.997, *f*_A_^ref^ = 0.769, *f*_H_^ref^ = 0.828.

The p*K*_a_ equation is treated similarly as in the prior example: p*K*_a_^*I*^ = 9.62 (slightly different from 9.60 due to the use of salting-out constant). The solubility equation in cumulative form (*cf.* Eq. A2) at the local ionic strength is determined (Eq. A3) as log *β*_HA(s)_^*I*^ = log *β*_HA(s)_^ref^ + log (*f*_A_^*I*^/*f*_A_^ref^) + log (*f*_H_^*I*^/*f*_H_^ref^). With the above activity coefficients, log *β*_HA(s)_^*I*^ = 10.28 + log (0.997/0.769) + log (0.997/0.828) = 10.47. From this, p*S*_0_^*I*^ = 10.47-9.62 = 0.85 (140 mM), which is only slightly lower than p*S*_0_^ref^ by 0.02 (=-*K*_salt_(*I-I*_ref_)).

### Example of the ‘constant ionic medium’ activity scale treatment in pH-adjusted saturated solution

As a further example drawing on analytic continuation of the above case, consider that 0.5 M HCl is added so that the pH is lowered to 0.05 (p_a_H). The activity coefficients of the various species need to be adjusted accordingly. So, at the target (local) pH, the ionic strength increases to *I* = 0.5 M. The second two terms in Eq. A4 take on more significance in the high ionic strength case. Using the approach presented above, *f*_A_^*I*^ = 0.743, *f*_A_^ref^ = 0.822, *f*_H_^*I*^ = 0.888, *f*_H_^ref^ = 0.885, and *f*_HA_^*I*^ = 1.144, *f*_HA_^ref^ = 1.041. These values substituted into Eq. 1 indicate that the p*K*_a_ value at *I* = 0.50 M becomes 9.33; that is, higher salt concentration makes the weak acid slightly stronger. The p*S*_0_^*I*^ at pH 0.05 (Eq. (3)) now increases from the value of 0.87 at pH_w_ 5.28 to



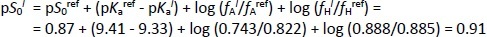

(3)


**Figure 1.**
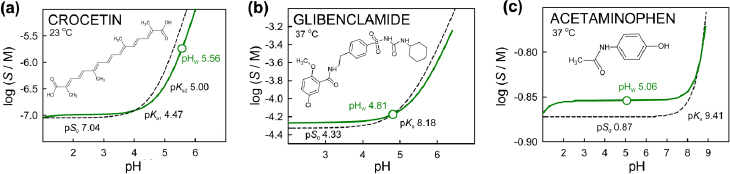
Profiles of log *S*-pH generated by analytic continuation for three weak acids, **(a)** crocetin [18], **(b)** glibenclamide [17], and **(c)** acetaminophen [17]. The solubility-pH curves (solid green) were generated by simulations started at pH_w_ with pH adjusted downwards using HCl titrant and upwards using NaOH. The dashed curves were calculated using the HH equation, incorporating p*K*_a_^ref^ and log *S*_0_^ref^ values, at *I*_ref_ = 0.15 M (without activity corrections)

Thus, at pH 0.05, the solubility (solid curve in Fig. 1c) crosses the HH curve (140 mM: dashed curve in Fig. 1c) as the solubility in the higher ionic strength suspension drops to 122 mM.

In this manner, at every pH point, a different set of p*K*_a_^*I*^ and p*S*_0_^*I*^ need to be determined, from which the local concentrations of reactants and product species are then calculated.

## Results and discussion

### Ionization constants

Table 1 lists pairs of p*K*_a_ values (p*K*_a_^ref^ and p*K*_a_^*I*^) of the 32 weakly ionizable molecules considered in the study. The listed temperature is that of the corresponding solubility study. In the solubility measurements in pure water, the benchmark reference ionic strength was selected as *I*_ref_ = 0.15 M. The local values of ionic strength, *I*_w_ (calculated at pH_w_ ), are listed. Values of p*K*_a_^ref^ are taken from published sources [28-34]; the corresponding p*K*_a_^*I*^ (at pH_w_) were calculated using *p*DISOL-X (*cf.* Eqs. (1) and (2).

### Solubility data and constants

Tables 2-4 summarize the details of the analyses of the published weak acid, base, and ampholyte solubility measurements in pure water. The literature values of log *S*_w_, along with the corresponding p*K*_a_ values (Table 1), were the starting points in the determination of pH_w_ and log *S*_0_^ref^. Activity corrections (Eqs. (1) to (3), based on the general Eq. A3 in Appendix A) were applied to ionic species. In addition, *K*_salt_ salting-out constants (Eq. (3) based on Eq. (A5)) were used to adjust the activities of uncharged species. In most cases in the tables, the *I*_w_ value at pH_w_ is near zero, particularly in the cases of weak bases, but the values increase significantly with the benzene carboxylic acid series, reaching a high value of 0.49 M for mellitic acid (2-fold excess added solid) or 0.38 M for mellitic acid (10-fold excess). Higher yet, lysine at pH_w_ indicates *I*_w_ = 0.61 M (1.1-fold excess) or *I*_w_ = 0.70 M (2-fold excess).

**Table 3.** Analysis results of solubility of ionizable bases in water (*I*_ref_ = 150 mM)

Weak bases	log (*S_w_*^Lit^ / M)	*S*_w_^Lit^ / mg mL^-1^	*t* / °C	Ref.	*K*_salt_ / M^-1^	pH_w_	log (*S*_0_^ref^ / M)	log (*S*_0_^*I*^ / M)	*I* / mM
Atenolol	-1.09	22	37	[26]	0.24	10.82	-1.13	*-1.09*	1.7
Bedaquiline	-5.62	0.0013	23	[21]	0.58	8.32	-6.25	*-6.16*	0.002
Brigatinib	-1.73	11	23	[25]	0.58	10.16	-1.82	*-1.73*	0.12
Carvedilol	-4.10	0.032	37	[17]	0.37	8.62	-4.21	*-4.16*	0.009
Emtricitibine	-0.38	104	25	[19]	0.14	8.15	-0.40	*-0.38*	0.001
Imatinib	-4.69	0.010	23	[24]	0.46	8.65	-4.85	*-4.78*	0.004
Imiquimod	-4.60	0.0061	25	[23]	0.28	7.97	-4.65	*-4.61*	0.001
Ketoconazole	-4.82	0.0080	37	[17]	0.50	7.48	-4.92	*-4.84*	0.001
Lamotrigine	-2.87	0.34	37	[17]	0.22	8.01	-2.91	*-2.88*	0.002
Lumefanatrine	-5.28	0.0028	23	[22]	0.64	8.71	-6.05	*-5.96*	0.004

**Table 4.** Analysis results of solubility of ampholytes in water (*I*_ref_ = 150 mM)

Ampholytes	log (*S_w_*^Lit^ / M)	S_w_^Lit^ / mg mL^-1^	*t* / °C)	Ref.	*K*_salt_ / M^-1^	pH_w_	log (*S*_0_^ref^ / M)	log (*S*_0_^*I*^ / M)	*I /* mM
Celecoxib	-5.10	0.003	37	[17]	0.34	6.71	-5.16	-5.11	0.0002
Enrofloxacin	-3.08	0.30	37	[17]	0.31	7.04	-3.22	-3.18	0.090
Lysine, L-^a^	0.60	585	27	[27]	0.10	10.01	0.53	0.47	695
Lysine, L-^b^						9.98	0.50	0.45	612
Mycophenolate mofetil	-2.93	0.51	37	[17]	0.51	7.06	-3.03	-2.96	0.038
Piroxicam	-4.02	0.032	37	[17]	0.20	4.77	-4.14	-4.11	0.019
Sulfamethoxazole	-3.36	0.11	37	[17]	0.20	4.68	-3.42	-3.39	0.024

^a^Total added 1.2 g / mL (2-fold excess over solubility value)

^b^Total added 0.6 g / mL (1.1-fold excess).

In almost all the simple acids, bases, and ampholytes, the measured solubility was slightly higher than the value harmonized to *I*_ref_, *i.e.* the presence of salt lowers water solubility at *I*_ref_ due to activity effects. In the case of lysine, the presence of salt elevates the solubility in water. These trends will be graphically illustrated below for a few of the substances studied.

The average values (± standard deviations) of salting-out coefficients, *K*_salt_ (Tables 2 to 4, estimated using Eq. (A5b)), are 0.28 ± 0.14 (ampholytes), 0.29 ± 0.13 (acids, first 8 in Table 2), and 0.40 ± 0.17 M^-1^ (bases). The average values are notably lower at 0.06 ± 0.06 for the benzene polycarboxylic acid series, particularly in the cases of three or more carboxylic acid substituents.

### Salting-out contributions to shifts in intrinsic solubility at different levels of ionic strength

At pH_w_, salting-out accounts for 92 to 100 % of the total activity correction for intrinsic solubility (*i.e.* log *S*_0_^ref^ - log *S*_0_^*I*^ ≈ *K*_salt_(*I*_ref_ - *I*)) and Eq. A3 does not contribute to the shift in log *S*_0_ between the measurement ionic strength and the reference 0.15 M value, since the second and third terms in Eq. (A3) are not significant at these levels of ionic strength.

### Solubility-pH curves generated by analytic continuation from pH_w_

Figure 1 illustrates the log *S*-pH relationships in three selected examples of acids (shown in increasing order of solubility): crocetin, glibenclamide and acetaminophen. At pH_w_, the ionic strength and buffer capacity are at their minimum values (with *I*_w_ ~0 M, buffer capacity < 1 mM/pH) for the three acids. As pH is altered by the addition of titrant, both properties increase, particularly below pH 3 and above pH 8.

In all cases in Figure 1, the equilibrium pH_w_ is less than that of water (pH ~7). Both the water solubility (*S*_w_) and the acid strength (p*K*_a_) of the molecule play a critical role, as detailed in the examples in Appendix C. Consider the weak acids in the figure. To better understand the resulting shifts from pH ~7 to pH_w_, it is helpful to think of the weak acid as a titrant added to pure water that produces a certain shift in water pH. For the most soluble of the acids in Figure 1, acetaminophen, the pH_w_ is shifted *below* the p*K*_a_ by 4.3 units. Since acetaminophen is quite soluble, enough of the free acid dissolves and sufficiently ionizes, to overcome the buffering of pure water in the neutral pH domain. In simulation calculations, acetaminophen, when added to water, releases ~0.08 μmol of H^+^ per mL of solution, which results in the pH shift from ~7 to ~75, well inside the minimum solubility region (pH <7, as dictated by the acetaminophen p*K*_a_) in the log *S*-pH profile (Fig. 1c). The lower the solubility of the acid, the less the acid impacts on the shift in pH_w_ from pH ~ 7 of pure water. For example, crocetin would need to release ~0.73 μmol/mL of H^+^ to effect the pH shift from ~7 to ~3, where the molecule would be in the intrinsic (minimum) solubility part of the log *S*-pH curve (Fig. 1a). However, due to its very low solubility, only about 0.06 μmol/mL of H^+^ are released, so crocetin cannot reach the intrinsic solubility region in the log *S*-pH profile (pH <3, as defined by its two p*K*_a_ values). The measured pH_w_ values are apt to be in the diagonal region of the profile, as in the case of the poorly soluble crocetin, where pH_w_ is above p*K*_a1_ by 0.94 log unit. By comparison, the pH_w_ of the more soluble glibenclamide is below its p*K*_a_ by 0.43 log unit.

In Figure 1c, the solid curve has a ‘rotated sigmoidal’ distortion, compared to the benchmark HH hyperbolic (dashed) curve. The solubility difference between the two curves maximizes at pH_w_ (where *I* and *I*_ref_ are most different). The solid and dashed curves cross at pH near 0.9 and 8.5, as the ionic strengths become equal at those points.

**Figure 2.**
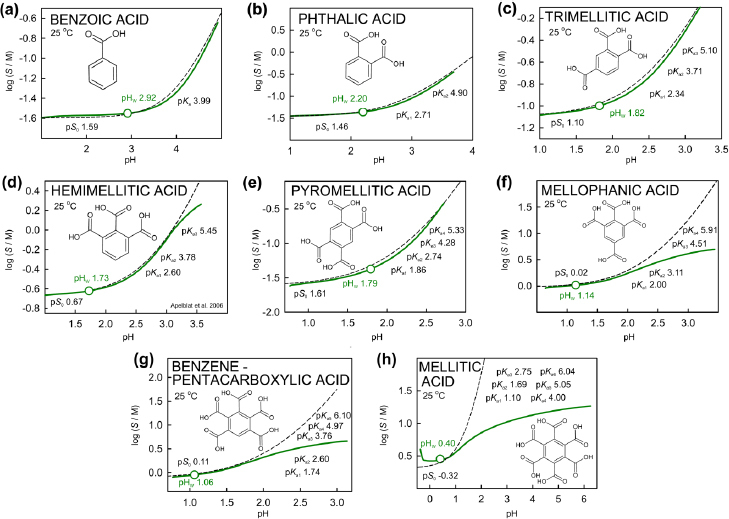
Calculated log S-pH profiles of the benzene polycarboxylic acid series [16]. The solubility-pH curves (solid green) were generated with simulations started at pH_w_ with pH adjusted downwards using HCl titrant and upwards using NaOH. The dashed curves were calculated using the HH equation, incorporating p*K*_a_^ref^ and log *S*_0_^ref^ values, at *I*_ref_ = 0.15 M (without corrections for activity effects)

Figure 2 shows log *S*-pH plots for the benzene polycarboxylic acid series. For benzoic, phthalic, trimellitic, and pyromellitic acids, the hyperbolic profiles take on typical activity-distorted shapes. As the number of carboxylic groups (with overlapping p*K*_a_) increases, so does the solubility, with mellitic acid reaching the high value of 988 mg/mL (Table 2). Due to the increasing ionic strength with solubility, especially as the charges increase on the polycarboxylates as pH increases, the distortions from the Henderson-Hasselbalch benchmarks (dashed curves in Fig. 2) become most pronounced. As pH increases, the increasing ionic strength exceeds 7 M past pH 2.3, 3.7, 4.1, and 7.0 for mellitic, benzene pentacarboxylic, mellophanic, and hemimellitic acids, respectively. The other four (mostly less soluble) carboxylic acids have ionic strengths that level off at pH 7.4 at *I* = 4.5 (trimellitic), 3.6 (pyromellitic), 1.2 (phthalic), and 0.3 M (benzoic).

The extreme distortions in Figure 2 related to activity corrections appear not to have been previously reported. In the case of mellitic acid, *I*_w_ exceed *I*_ref_, and depends on the excess quantity of neutral acid solid added to distilled water (*cf.* Table 1). This concentration dependence is also evident in the case for L-lysine. From the perspective of solubility measurement, when *I*_w_ exceeds *I*_ref_, it may be of limited use to adjust the water solubility observed at *I*_w_ to the expected value at the physiologically relevant *I*_ref_, since at that reference ionic strength, both mellitic acid and L-lysine are apt to dissolve completely at practical concentrations.

**Figure 3.**
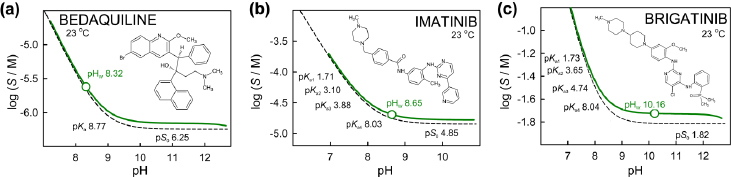
Simulated log *S*-pH profiles of three weak based: (a) bedaquiline [21], (b) imatinib [24] and (c) brigatinib [25], ordered by increasing intrinsic solubility

Figure 3 illustrates the log S-pH relationships of three selected bases (arranged in increasing order of intrinsic solubility). The ionic strength values are at their minimum at pH_w_. As pH is adjusted by additions of HCl/NaOH in the simulated analytic continuation procedure, the ionic strength increases, just as in the cases of simple weak acids. The characteristic distortions of the solid (green) curves are the consequence of activity corrections, which depend on the differences, *I* - *I*_ref_. A partial ‘rotated sigmoidal’ shape distortion is evident for bedaquiline and brigatinib, compared to the HH hyperbolic dashed curve at *I*_ref_ = 0.15 M.

The position of pH_w_ in relation to the p*K*_a_ mirrors the trends illustrated in Figure 1 and can be explained by the effects of solubility and how far the p*K*_a_ values are from pH ~7 of pure water. The less soluble the base, the more is the pH_w_ apt to be in the diagonal region of the log S-pH profile.

**Figure 4.**
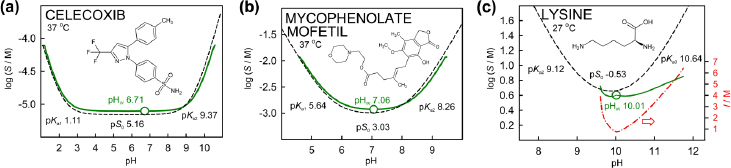
Simulated log S-pH profiles of three ampholytes: (a) celecoxib [17], (b) mycophenolate mofetil [17] and (c) L-lysine [27], ordered by increasing intrinsic solubility. The red dash-dot curve in frame (c) refers to the ionic strength as a function of pH

Figure 4 shows the log *S*-pH profiles of three selected ampholytes, arranged in increasing order of intrinsic solubility. The differences in the curves between the low ionic strengths (solid green) and the reference level (dashed) curves in the first two examples may be anticipated based on the preceding acid-base discussions.

Except for lysine, the ampholytes (Table 4) show positive log *S*_0_^*I*^ - log *S*_0_^ref^ differences of 0.029 to 0.051 (less soluble with increased salt). Lysine appears to show negative (salting-in) differences of -0.048 (1.1-fold excess solid added) or -0.056 (2-fold excess) - more soluble with increased salt. This salting-in value appears to be entirely *K*_salt_ driven, with no contribution from the traditional SRHT Eq. A3. The dash-dot (red) curve in Figure 4c shows the asymmetric lysine ionic strength profile as a function of pH. Ionic strength values exceeding 7 M are reached as pH is altered from the pH_w_.

### Limitations in transforming S_w_ solubility in pure water to physiologically-appropriate solubility-pH

In the absence of high quality *S*_w_ solubility and p*K*_a_ measurement, the additional ‘fine tuning’ corrections for the ionic strength effect would hardly be worthwhile. The uncertainty in the conversion of *S*_w_ (typically at *I*_w_ ~ 0 M) to *S*_0_ at *I*_ref_ = 0.15 M and consequently extending that into a log *S*-pH reference-state profile probably has as much to do with the reliability of the measured *S*_w_ and the provided *I*_ref_ -based p*K*_a_ values, as with the modified SRHT model described here.

The following shortfalls underlie the challenge in validating the conversion procedure we discussed:

As part of the *S*_w_ assay, measurement of the pH_w_ would be helpful, as this would allow for recognition/correction of the effects of impurities (*e.g.* CO_2_) and could lead to more certain *S*_w_ measurement.

- Specified electrode calibration/standardization (*e.g.* junction potential determined from blank titrations - see Appendix B) would allow for the confident conversion of operational pH (p_a_H) to concentration-based p_c_H, so constants could be reported consistently at *the ‘*constant ionic medium’ activity scale. This would be particularly important if pH_w_ were measured in very acidic/basic solutions.
- For relatively soluble drugs, the *actual weight* of solid added to produce a saturated solution would be helpful to report, since the conversion process may depend on the excess solid added (*e.g.* mellitic acid in Table 2, lysine in Table 4).
- For very soluble molecules, the added amount of solid would be expected to change the total solution volume. Measurement or estimation of density of the suspension (*e.g.* applying McGowan molar volume) would allow the conversion of concentrations from molality to molarity units.
- The hydration numbers, *h*_j_ (*i.e.* the number of water molecules sequestered in the solvation shell of the j^th^ species; *cf.* Appendix A), in the SRHT model for charged and uncharged solutes are only tentatively defined for druglike molecules, which contributes to the uncertainty in the SRHT model [14].
- Experimental activity coefficients are scarcely available for druglike molecules, to test reliability of the modified SRHT model.
- An actual measurement of a log *S*-pH profile covering a range of pH would be a helpful test of the reliability of the conversion process described in this commentary, particularly in the case of lysine. Usually, such measurements are done in buffered solutions, rather than simply in water. We have not been able to find *reliable* published results of lysine solubility as a measured function of pH in unbuffered solutions. The lysine log S-pH data from Amend and Helgeson [56] are inconsistent with the *S*_w_ value reported by Dooley and Castellino [27]. Nor is the shape of the curve [56] consistent with the value of p*K*_a3_. Zhang *et al.* [57] reported a measured value of *S*_w_ of lysine which is in good agreement with that of Dooley and Castellino. However, solubility values at pH other than pH_w_ were not directly measured. Zhang *et al.* [57] used a functional-group activity coefficients (UNIFAC) model to calculate the log S-pH profile. So, validation with *measured* values of log S over the pH range of interest would be a welcome contribution.

## Conclusion

Published values of neutral drug solubility in pure water, *S*_w_, can be quite useful for ranking research compounds according to solubility. All too often, the published assay details are sparsely specified, making quality assessment of the reported solubility challenging. So, from a solution chemistry mechanistic point of view, the application of *S*_w_ can be problematic. For newly-approved drugs, usually, only simple water solubilities are publicly available, as gleaned from NDA filings and/or patents.

However, there are opportunities to extract potentially useful additional information from such measurements. For example, the reliability of the measured *S*_w_ would be confirmed if the corresponding pH_w_ of the saturated solutions were measured and reported. Perhaps the reporting of measured pH_w_ will be more common, as improved understanding of the broader applicability of *S*_w_ is recognized more widely.

This commentary describes known but often neglected *steps* which could be taken, based on rigorous activity corrections, to draw out valuable information about the solubility of druglike substances as a function of pH and ionic strength when only a single-point *S*_w_ is reported. Examples employing thirty-two critically selected weakly-ionizable molecules form the springboard for the methods reviewed.

When a weakly ionizable molecule in neutral form is added to pure water in sufficient amount to form a saturated solution, the equilibrium pH is reached at pH_w_. The ionic strength, *I*_w_, is close to *zero* at that pH, unless the molecule is very soluble and/or is polyprotic. Also, the buffer capacity is often at a local minimum at pH_w_ (barely so in the interesting case of lysine). If a strong acid/base is used to adjust the pH away from pH_w_, the ionic strength increases, substantially in some cases (*e.g.* with lysine, *I* > 10 M at pH 7.4).

The physiologically relevant ionic strength is close to 0.15 M. To address the effects of shifts in ionic strengths, a rigorous method incorporating activity coefficients into the equilibrium constants based on the Stokes-Robinson hydration theory is discussed at length in Appendix A, with explicit examples given in the main text of the commentary. This includes using estimated ‘salting-out’ constants to adjust activity coefficients of neutral species.

In cases where experimental pH_w_ values are not reported (or even measured), a completely general computational method for determining the water pH_w_ for saturated solutions of neutral drugs is described here (assuming impurity-free suspensions). For monoprotic molecules, simple explicit equations are presented in Appendix C. For diprotic acids and bases, polynomial equations in [H^+^] taken to the power of three are derived. The latter can be solved using a simple spreadsheet method [52]. Apparently, for triprotic and higher order molecules, explicit equations have not been described in the literature. The *p*DISOL-X program does not rely on the explicit equations listed in the Appendix and elsewhere [12] but rather develops internal implicit mass action equilibrium casting to calculate pH_w_. Nevertheless, the explicit equations can be useful for checking the *p*DISOL-X calculation results for the simpler mono- and diprotic molecules.

Analytic continuation is a mathematical procedure, where given all the relevant equilibrium constants and total concentrations, the entire solubility profile can be simulated for a wide range of pH. Such log *S*-pH curves may be harmonized to any practical level of ionic strength, well above the near zero values characteristic of the measurements of *S*_w_.

Since activity coefficients depend on the differences between the low ionic strength in the measurement and the elevated physiologically-relevant reference level, the shape of the log S-pH curve generated by analytic continuation can be distorted, compared to the hyperbolic shaped curve generated by the benchmark Henderson-Hasselbalch equation. Acetaminophen is an example of a small ‘rotated sigmoidal’ shape distortion (solid green curve, Fig. 1c). In the cases of benzene tri- to hexa- carboxylic acid derivatives (Fig. 2d, 2f, 2g and 2h) and lysine (Fig. 4c), distortions of the solubility-pH curves generated by analytic continuation take on forms which show considerable departure from those indicated by the Henderson-Hasselbalch equation. This is a novel prediction, which still needs to be experimentally validated by solubility measurements at pH values apart from pH_w_.

A partial test of the effectiveness of the SRHT model can be made by directly determining the activity coefficients of hydrogen ions using the approach in Appendix B. Also, selecting compounds whose p*K*_a_ values are reliably known as a function of ionic strength (*e.g.* phthalic acid) could be used to compare the predicted shifts in p*K*_a_ values based on the SRHT model to those extracted from known p*K*_a_ values. It would be an ongoing process of refining the SRHT model, to improve its effectiveness.

It is stressed that transforming measured drug solubility in water to physiologically-appropriate solubility-pH would better match the conditions found in biological media, potentially improving applications of solubility in pharmaceutical research and development.

## Supplementary material

Additional data are available at https://pub.iapchem.org/ojs/index.php/admet/article/view/2626, or from the corresponding author on request.


