# Supplementary material for: Often neglected steps in transforming drug solubility from single measurement in pure water to physiologically-appropriate solubility-pH
Source: ADMET DMPK. 2025 Feb 26;13(1):2626. doi: 10.5599/admet.2626 (PMC11954141; doi:10.5599/admet.2626)
Supplement: Supplementary file 1 [file ADMET-13-2626-SM.pdf]

ADMET & DMPK 13(1) (2025) S2626

Open Access : ISSN : 1848-7718

http://www.pub.iapchem.org/ojs/index.php/admet/index

Supplementary material to

Often neglected steps in transforming drug solubility from single measurement in pure water to physiologically-appropriate solubility-pH

Alex Avdeef

in-ADME Research, New York City, NY 10128, USA

ADMET & DMPK **13(1)** (2025) 2626; https://doi.org/10.5599/admet.2626

The Appendix describes several features of *p*DISOL-X that potentially extend the program's application reach to the high values of ionic strengths (I > 5 M). The use of the Stokes-Robinson hydration theory (SRHT) is briefly reviewed here. In *p*DISOL-X calculations, equilibrium constants are automatically compensated for changes in ionic strength resulting during a pH titration, using SRTH, which extends the simple Debye-Hückel equation to I > 0.1 M. Also, the Appendix includes sample derivations of *explicit* solubility-pH equations for two simple cases of acids and bases, and for a typical diprotic ampholyte.

It is assumed here that a specified weight of a neutral substance (HA, B, or XH) is added to a volume of distilled water, at an amount enough to form a saturated solution across a wide pH range when pH is adjusted with a simple titrant, HCl or NaOH. Under these circumstances, the pH of the initial equilibrated solution is expected to be at the saturation endpoint, pH_w, assuming the solution is free of ambient carbon dioxide and that ancillary complexation/aggregation is not an interfering factor. A simulated solubility-pH profile is generated as acid-base titrants are then added.

Appendix A. Automatic ionic strength compensation

Unless intentionally controlled, the ionic strength, I, changes during an acid-base solubility-pH titration due to ionizations, additions of titrant, and dilution effects. This change affects equilibrium constants. In many cases, uncontrolled ionic strength can vary substantially during titration. In contrast, standardized methods for pK_a determination are typically conducted at a nearly constant I_{ref} = 0.15 M (adjusted with KCl or NaCl), under conditions where low sample concentrations (*e.g.* 10^{-3} to 10^{-6} M) are 'swamped' by the added inert salt.

In the 'constant ionic medium' model, it is a preferred practice to designate 0.15 M as the 'reference' ionic strength, I_{ref} ('physiological' level), to which calculated equilibrium constants are adjusted at each pH point in the solubility assay calculations. There is no loss of thermodynamic rigor over the legacy practice of defining the reference state at zero concentration and unit activity coefficients [1] (pp. 43-47).

Since I at any given pH point is likely different from I_{ref}, all ionization constants need to be *locally* transformed (from reference I_{ref} to local I) for the calculation of local point concentrations.

Consider a two-reactant system, based on reactants A (*e.g.* anion of weak acid) and H (proton), whose charges are Q and +1, respectively. The concentration of the j_{th} species, C_j, is calculated in terms of these reactants; Eq. (A1)

$$C_j = C_{A^Q}{}^{E_{aj}} \, C_{H^+}{}^{E_{hj}} \, \beta_j \tag{A1}$$

defined by the general equilibrium Eq. (A2)

$$E_{aj} \text{ A} + E_{hj} \text{ H} \leftrightarrows \text{A}_{E_{aj}} \text{H}_{E_{hj}} \tag{A2}$$

where β_j is the 'cumulative' formation constant [1] (pp. 148-157), and E_{aj} and E_{hj} are the A and H stoichiometric coefficients, respectively, of the jth species. The core idea here is to place only the 'bare' reactants on the left side of the equilibrium expression and the product species formed on the right side.

The $\beta(I_{ref})$ reference set of cumulative constants (*i.e.* those refined by regression analysis) are transformed to the local set $\beta(I)$ according to the general Eq. (A3)

$$\log\beta_j(I) = \log\beta_j(I_{ref}) + E_{aj}\log\left[\frac{f_A(I)}{f_A(I_{ref})}\right] + E_{hj}\log\left[\frac{f_H(I)}{f_H(I_{ref})}\right] - \log\left[\frac{f_j(I)}{f_j(I_{ref})}\right] \tag{A3}$$

The ionic-strength-dependent activity coefficients of A, H, and jth species are denoted f_A, f_H, and f_j, respectively. The activity coefficients are cast in an expanded equation based on the hydration theory proposed by Robinson and Stokes [2,3], further elaborated by Bates *et al.* [4] and Robinson and Bates [5] to include single-ion activities, then slightly modified by Bockris and Reddy [6] and recently applied to solubility data of a druglike molecule by Wang *et al.,* Eq. (A4) [7]:

$$\log f_i(I) = -Q_i^2 \, \frac{A\sqrt{I}}{1 + B\mathring{a}_i\sqrt{I}} - h_i \log a_{H_2O} + \log\left(\frac{C_{H_2O} + \sum_j C_j}{C_{H_2O} + \sum_j (1-h_j)C_j}\right) \tag{A4}$$

The first term on the right side of Eq. (A4) is the Debye-Hückel equation accounting for the long-range ion-ion electrostatic interactions. At 25 °C and I = 0.0, 0.15, and 1.0 M (NaCl), the respective parameters (molar scale) are: $A = 1.825\times10^6\,(\varepsilon T)^{-3/2}$ = 0.512, 0.528, 0.642; ion size scaler, $B = 50.29\,(\varepsilon T)^{-1/2}$ = 0.329, 0.333, 0.355; dielectric constant of water, ε = 78.3, 76.8, 67.3. T / K is the absolute temperature. The mean diameter of the ith hydrated ion is $\mathring{a}_i$ [8].

The second and third terms are ascribed to short-range ion-solvent interactions. The second term is related to the decrease in the activity of water due to the work done in immobilizing some of the bulk water to hydrate ions. The activity of water, a_{H_2O} = 1.000, 0.995, 0.967 [6] at the above three ionic strength values. The last term in Eq. A4 is related to free energy change of the ions, as their concentrations effectively increase when the 'available' volume of bulk water decreases upon hydration of the ions. The summation symbols in Eq. (A4) are over all charged species (including the reactants, inert salt and buffer components) under consideration. The concentration of pure water is C_{H_2O} = 55.51 M. The h_j in the summation term is hydration number of the jth ion [3]. Values of h_j were selected/estimated as described by Wang *et al.* [7]. For neutral molecules, it is often assumed that h_j = 0.

The activity coefficients of uncharged species can be determined by the second two terms in Eq. (A4). In practice, the activity coefficients are barely different from 1. As a modification to Eq. A4, in *p*DISOL-X, the activity of uncharged species (*e.g.* HA...H_6A, B, XH) includes the contribution of the salting-out factor (Setschenow constant [9]), K_{salt} / M^{-1} , the values of which were estimated empirically using Eq. (A5b) [10].

$$\log f_s = K_{salt}(I - I_{ref}) \tag{A5a}$$

CC BY

$$K_{salt} = 0.090 - 0.073A - 0.064B - 0.039S_\pi - 0.002E + 0.188V \qquad (A5b)$$

A, B, S_π, E and V are the five Abraham solvation descriptors: H-bond acidity, H-bond basicity, dipolarity-polarizability, excess molar refractivity, and McGowan molar volume, respectively [11,12]. In the calculation of activity coefficients in *p*DISOL-X, all charged species are treated with the legacy SRHT Eq. (A4). However, if a neutral solute is considered, then Eq. (A5a) is used to calculate its activity coefficient. All solids are assigned $\log f_s = 1$.

Appendix B. pH electrode calibration, standardization and automatic compensation

The 'blank' acid-base titration method can be used, based on an empirical four parameter equation, in what has been called the 'standardization' step [13], Eq (A6).

$$pH = \alpha + k_S\, p_cH + j_H C_{H^+} + j_{OH} K_w / C_{H^+} \qquad (A6)$$

where pH is the 'operational' pH (meter reading) and K_w is the ionization constant of water, which is a function of temperature and ionic strength [14]. The j_H term corrects pH readings for the nonlinear pH response due to liquid junction and asymmetry potentials in highly acidic solutions (pH <2), while the j_{OH} term corrects for high-pH nonlinear effects [1] (pp. 140-145). Typical values of the adjustable parameters at 25 °C, based on titration of 'blank' aqueous solutions containing 0.15 M KCl, are $\alpha = 0.09$, $k_S = 1.002$, $j_H = +0.5$ and $j_{OH} = -0.5$. However, each electrode possesses its own characteristic set, which can shift with repeated exposure to saturated solutions.

In solubility-pH profiles, ionic strength may reach values exceeding 10 M. The experimentally determined parameters in Eq. (A6) are automatically compensated in the data analysis program, for changes in I at the local pH from the benchmark level of I_{ref} = 0.15 M, according to empirically determined relationships [1] (pp. 64-65), Eqs. (7a) to (7d):

$$\alpha = 0.059 + 0.085 \frac{\sqrt{I}}{(1+\sqrt{I})} \qquad (A7a)$$

$$k_S = 0.995 + 0.032\left(\frac{\sqrt{I}}{1+\sqrt{I}} - 0.57I\right) \qquad (A7b)$$

$$j_H = 0.80 - 2.0I \qquad (A7c)$$

$$j_{OH} = -0.59 + 0.6I \qquad (A7d)$$

Appendix C. Explicit solubility-pH equations in saturated solution

All the equations derived below refer to aqueous solutions saturated with a neutral weak acid (HA), a weak base (B), or an ampholyte (XH). To keep the equations simple and relatively transparent, in the following sample derivations of explicit equations, activity and salting-out corrections are not applied in the derivation of the saturation endpoint pH_w. All total reactant concentrations are taken to be less than the salt solubility, so only the neutral species can precipitate as excess solids. The concentrations of the neutral species are frequently denoted as $C_{HA\,s}$, $C_{B\,s}$ and $C_{XH\,s}$, to emphasize that these species are saturated in the solutions. The subscripts 's' will not be used here, since the defined context here is unambiguous. The lowest possible solubility value of the neutral species is called the 'intrinsic solubility', denoted by the symbol S_0.

Saturated solution of monoprotic weak acid, HA, in pure water

The equilibrium 'model' refers to a set of equilibrium equations and the associated equilibrium constants. In the case of a monoprotic weak acid, a saturated solution can be defined by the two equations and the corresponding constants

$HA \leftrightarrows H^+ + A^-$ $\quad K_a = C_{H^+} C_{A^-} / C_{HA}$ (A8a)

$HA(s) \leftrightarrows HA$ $\quad S_0 = C_{HA}$ (A8b)

K_a is the ionization constants of the weak acid and S_0 is the intrinsic solubility of the acid. The solubility, S, at a particular pH is defined as the mass balance sum of the concentrations of all the A-containing components dissolved in the aqueous phase, $A_{tot,aq}$, Eq. (A9)

$$S = C_{HA}+C_{A^-} = C_{HA} + K_a\, C_{HA}/ C_{H^+} = S_0\,(1 + K_a / C_{H^+}) \quad \text{(A9)}$$

The square brackets denote molar concentration of species. In logarithmic form, Eq. (A10)

$$\log S = \log S_0 + \log (1 + 10^{-\,pK_a + pH}) \quad \text{(A10)}$$

Eq. A10 is called the Henderson-Hasselbalch (HH) equation for a monoprotic weak acid. The plot of log S *vs.* pH is defined by a hyperbolic curve. In the plot, the limiting slope in acidic solutions (pH << pK_a) is zero and in alkaline solutions (pH >> pK_a) is +1. pK_a is indicated by the pH where the slope is +½.

To calculate the equilibrium pH$_w$, it is useful to start with the total hydrogen excess in aqueous solution, $H_{tot,aq}$, expressed in terms of the equilibrium model (Eq. (A8)) and set equal to the analytical acid-base concentrations (C_{HCl}, C_{NaOH} and $A_{tot,aq}$):

$$H_{tot,aq} = C_{H^+} - C_{OH^-} + C_{AH} = C_{H^+} - K_w/C_{H^+} + S_0 = C_{HCl} - C_{NaOH} + A_{tot,aq} = C_{HCl} - C_{NaOH} + S_0 + C_{A^-} \quad \text{(A11)}$$

On rearranging the above, noting that at endpoint pH$_w$, $C_{HCl} = C_{NaOH}$, EQs. (12)

$$C_{H^+} - K_w/C_{H^+} + S_0 = C_{HCl} - C_{NaOH} + S_0 + C_{A^-} \quad \text{(A12a)}$$

$$C_{H^+} - K_w/C_{H^+} - C_{A^-} = 0 \quad \text{(A12b)}$$

$$C_{H^+} - K_w/C_{H^+} - S_0K_a / C_{H^+} = 0 \quad \text{(A12c)}$$

$$C_{H^+}{}^2 = K_w + S_0K_a \quad \text{(A12d)}$$

The equilibrium pH of a saturated solution of a monoprotic weak acid is

$$(C_{H^+w})^{ACID} = (K_w + S_0\, K_a)^{1/2} \quad \text{(A13)}$$

Saturated solution of diprotic weak acid, H_2A, in pure water

The above procedure can be extended to the case of a diprotic acid, although C_{H^+w} is no longer easily stated in explicit form. As such, the equation for the total hydrogen excess in aqueous solution is given by Eq. (A14)

$$H_{tot,aq} = C_{H^+} - C_{OH^-} + C_{AH^-} + 2\, C_{H_2A} = C_{HCl} - C_{NaOH} + 2A_{tot,aq} \quad \text{(A14)}$$

Eq. (A14) is then transformed by inserting the expressions for pK_{a1}, pK_{a2} and S_0. At the saturation endpoint, $C_{HCl} = C_{NaOH}$. The transformed equation is further re-arranged to the cubic formula in terms of C_{H^+w}.

$$(C_{H^+w})^3 - C_{H^+w}\,(K_w + K_{a1}S_0) - 2\, K_{a1}K_{a2}\, S_0 = 0 \quad \text{(A15)}$$

The equation may be readily solved using a spreadsheet method, as described elsewhere [15]. For triprotic and more complicated multiprotic weak acids, the derived higher-order polynomial equations analogous to the above expression become unwieldy. Their exact forms appear not to have been published. Rather than dealing with explicit equations (*e.g.* Eqs. (A13), (A15), (A19), (A20), (A25)), the *p*DISOL-X program automatically derives implicit equivalents, and readily adapts them to activity corrections.

Saturated solution of monoprotic weak base, B, in pure water

In the case of a monoprotic weak base, a saturated solution can be defined by Eqs. (16)

$BH^+ \leftrightarrows H^+ + B$ $\quad K_a = C_{H^+}C_B / C_{BH^+}$ (A16a)

$B(s) \leftrightarrows B$ $\quad S_0 = C_B$ (A16b)

CC BY

The total solubility, S, at a particular pH is defined by Eq. (A17)

$$S = C_B + C_{BH^+} \quad (A17)$$

The above equation can be further transformed to Eq. (A18) as noted above.

$$\log S = \log \{C_B + C_B C_{H^+} / K_a\} = \log C_B + \log (1 + C_{H^+} / K_a) = \log S_0 + \log (1 + 10^{+pK_a - pH}) \quad (A18)$$

The above Henderson-Hasselbalch (HH) equation (A18) for a monoprotic weak acid describes a hyperbolic log S - pH curve, vertically mirroring that of the weak acid case. The limiting slope in acidic solutions (pH << pK_a) is -1 and in alkaline solutions (pH >> pK_a) is zero. The pK_a is indicated by the pH where the slope is -½.

Following similar steps noted for the case of a monoprotic weak acid, the corresponding explicit Eq. (A19) for the equilibrium pH can be derived for a monoprotic weak base as

$$(C_{H^+w})^{BASE} = \{K_w / (1 + S_0 / K_a)\}^{1/2} \quad (A19)$$

For a diprotic weak base, the corresponding explicit formula derives as the cubic Eq. (A20)

$$(C_{H^+w})^3 - (C_{H^+w})^2 K_{a1}(1 + K_{a2} / S_0) / 2 - K_w K_{a1} K_{a2} / S_0 = 0 \quad (A20)$$

As noted above, the equation may be readily solved using a spreadsheet method [15].

Saturated solution of diprotic ampholyte, XH, in pure water

A saturated ampholyte solution can be defined by the equations (A21) and the corresponding constants:

$$XH_2^+ \leftrightarrows H^+ + XH \qquad K_{a1} = C_{H^+} C_{XH} / C_{XH_2^+} \quad (A21a)$$

$$XH \leftrightarrows H^+ + X^- \qquad K_{a2} = C_{H^+} C_{X^-} / C_{XH} \quad (A21b)$$

$$HX(s) \leftrightarrows XH \qquad S_0 = C_{XH} \quad (A21c)$$

The total solubility, S, at a particular pH is defined as the mass balance sum of the concentrations of all the species dissolved in the aqueous phase:

$$S = C_{X^-} + C_{XH} + C_{XH_2^+} = X_{tot,aq} \quad (A22)$$

The above equation can be transformed into an expression containing only constants and C_{H^+} (as the only variable), by substituting the above ionization and solubility Eq. (A23).

$$\log S = \log \{K_{a2} C_{XH}/C_{H+} + C_{XH} + C_{XH}C_{H^+} / K_{a1}\} = \log C_{XH} + \log (K_{a2} / C_{H^+} + 1 + C_{H^+} / K_{a1}) =$$
$$= \log S_0 + \log (1 + 10^{-pK_{a2}+pH} + 10^{+pK_{a1}-pH}) \quad (A23)$$

The above HH equation for an ampholyte describes a U-shaped log S-pH curve. The limiting slope in acidic solutions is -1 and in alkaline solutions is +1. The limiting slope between the two pK_a values is zero. pK_{a1} is indicated by the pH where the slope is -½; pK_{a2} is indicated by the pH at slope +½.

As before, pH$_w$ can be calculated from the total hydrogen excess, $H_{tot,aq}$, expressed in terms of the equilibrium model (Eqs. (A21)) and set equal to the analytical acid-base concentrations (C_{HCl}, C_{NaOH} and $X_{tot,aq}$), Eq. (A24):

$$H_{tot,aq} = C_{H^+} - C_{OH^-} + C_{XH} + 2\, C_{XH_2^+} = C_{H^+} - K_w/C_{H^+} + S_0 + 2S_0 C_{H^+} / K_{a1} = C_{HCl} - C_{NaOH} + X_{tot,aq} \quad (A24)$$

On rearranging the above (noting that at endpoint pH$_w$, $C_{HCl} = C_{NaOH}$), the equilibrium pH of a saturated solution of a monoprotic weak acid is given by Eq. (A25)

$$(C_{H^+w})^{AB} = \{(K_w + S_0 K_{a2}) / (1 + S_0/K_{a1})\}^{1/2} \quad (A25)$$

References

[1] A. Avdeef. *Absorption and Drug Development, 2nd Ed.*, Wiley-Interscience, Hoboken NJ, 2012. ISBN 978-1-118-05745-2. https://doi.org/10.1002/9781118286067

[2] R.H. Stokes, R.A. Robinson. *Journal of the American Chemical Society* **70** (1948**)** 1870-1878. https://doi.org/10.1021/ja01185a065

[3] R.A. Robinson, R.H. Stokes. *Electrolytic Solutions*. 2nd Rev. Ed. Dover Publications, Inc., Mineola, NY. 2002, pp. 18-19. https://easypdfs.cloud/downloads/4879156-electrolyte-solutions-robinson-stokes

[4] R.G. Bates, B.R. Staples, R.A. Robinson. Ionic hydration and single ion activities in unassociated chlorides at high ionic strengths. *Analytical Chemistry* **42** (1970) 867-871. https://doi.org/10.1021/ac60290a006

[5] R.A. Robinson, R.G. Bates. Ionic activity coefficients in aqueous mixtures of NaCl and $MgCl_2$. *Marine Chemistry* **6** (1978) 327-333. https://doi.org/10.1016/0304-4203(78)90013-0

[6] J.O'.M. Bockris, A.K.N. Reddy. *Modern Electrochemistry*, Vol. 1. Plenum Publishing Corp., New York, NY, 1973. https://doi.org/10.1007/b114546

[7] Z. Wang, L.S. Burrell, W.J. Lambert. Solubility of E2050 at various pH: a case in which apparent solubility is affected by the amount of excess solid. *Journal of Pharmaceutical Sciences* **91** (2002) 1445-1455. https://doi.org/10.1002/jps.10107

[8] J. Kielland. Individual activity coefficients of ions in aqueous solutions. *Journal of the American Chemical Society* **59** (1937) 1675-1678. https://doi.org/10.1021/ja01288a032.

[9] N. Ni, S.H. Yalkowsky. Prediction of Setschenow constants. *International Journal of Pharmaceutics* **254** (2003) 167-172. https://doi.org/10.1016/S0378-5173(03)00008-5

[10] A. Avdeef. Anomalous salting-out, self-association and pK_a effects in the practically-insoluble bromothymol blue. *ADMET & DMPK* **11** (2023) 419-432. https://doi.org/10.5599/admet.1822

[11] M.H. Abraham, J. Le. The correlation and prediction of the solubility of compounds in water using an amended solvation energy relationship. *Journal of Pharmaceutical Sciences* **88** (1999) 868-880. https://doi.org/10.1021/js9901007

[12] J.A. Platts, D. Butina, M.H. Abraham, A. Hersey. Estimation of molecular linear free energy relation descriptors using a group contribution approach. *Journal of Chemical Information and Computer Sciences* **39** (1999) 835-845. https://doi.org/10.1021/ci980339t

[13] A. Avdeef, J.J. Bucher. Accurate measurements of the concentration of hydrogen ions with a glass electrode: calibrations using the Prideaux and other universal buffer solutions and a computer-controlled automatic titrator. *Analytical Chemistry* **50** (1978) 2137-2142. https://doi.org/10.1021/ac50036a045

[14] F.H. Sweeton, R.E. Mesmer, C.F. Baes, Jr. Acidity measurements at elevated temperatures. VII. Dissociation of water. *Journal of Solution Chemistry* **3** (1974) 191-214. https://doi.org/10.1007/BF00645633.

[15] A. Avdeef, K. Sugano. Salt solubility and disproportionation - uses and limitations of equations for pHmax and the in-silico prediction of pHmax. *Journal of Pharmaceutical Sciences* **111** (2022) 225-246. https://doi.org/10.1016/j.xphs.2021.11.017

CC BY
