# Supplementary material for: Often neglected steps in transforming drug solubility from single measurement in pure water to physiologically-appropriate solubility-pH
Source: ADMET DMPK. 2025 Feb 26;13(1):2626. doi: 10.5599/admet.2626 (PMC11954141; doi:10.5599/admet.2626)
Supplement: Supplementary file 2 [file ADMET-13-2626-S1.docx]

*ADMET & DMPK 13(1) (2025) S2626*

*
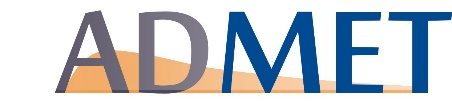
***Open Access : ISSN : 1848-7718**[***http://www.pub.iapchem.org/ojs/index.php/admet/index***](http://www.pub.iapchem.org/ojs/index.php/admet/index)

Supplementary material to

**Often neglected steps in transforming drug solubility from single measurement in pure water to physiologically-appropriate solubility-pH**

Alex Avdeef

*in-ADME Research, New York City, NY 10128, USA*

ADMET & DMPK **13(1)** (2025) 2626; <https://doi.org/10.5599/admet.2626>

The Appendix describes several features of *p*DISOL-X that potentially extend the program’s application reach to the high values of ionic strengths (*I* > 5 M). The use of the Stokes-Robinson hydration theory (SRHT) is briefly reviewed here. In *p*DISOL-X calculations, equilibrium constants are automatically compensated for changes in ionic strength resulting during a pH titration, using SRTH, which extends the simple Debye-Hückel equation to *I* > 0.1 M. Also, the Appendix includes sample derivations of *explicit* solubility-pH equations for two simple cases of acids and bases, and for a typical diprotic ampholyte.

It is assumed here that a specified weight of a neutral substance (HA, B, or XH) is added to a volume of distilled water, at an amount enough to form a saturated solution across a wide pH range when pH is adjusted with a simple titrant, HCl or NaOH. Under these circumstances, the pH of the initial equilibrated solution is expected to be at the saturation endpoint, pH_w_, assuming the solution is free of ambient carbon dioxide and that ancillary complexation/aggregation is not an interfering factor. A simulated solubility-pH profile is generated as acid-base titrants are then added.

Appendix A. Automatic ionic strength compensation

Unless intentionally controlled, the ionic strength, *I*, changes during an acid-base solubility-pH titration due to ionizations, additions of titrant, and dilution effects. This change affects equilibrium constants. In many cases, uncontrolled ionic strength can vary substantially during titration. In contrast, standardized methods for p*K*_a_ determination are typically conducted at a nearly constant *I*_ref_ = 0.15 M (adjusted with KCl or NaCl), under conditions where low sample concentrations (*e.g.* 10^-3^ to 10^-6^ M) are ‘swamped’ by the added inert salt.

In the ‘constant ionic medium’ model, it is a preferred practice to designate 0.15 M as the ‘reference’ ionic strength, *I*_ref_ (‘physiological’ level), to which calculated equilibrium constants are adjusted at each pH point in the solubility assay calculations. There is no loss of thermodynamic rigor over the legacy practice of defining the reference state at zero concentration and unit activity coefficients [1] (pp. 43-47).

Since *I* at any given pH point is likely different from *I*_ref_, all ionization constants need to be *locally* transformed (from reference *I*_ref_ to local *I*) for the calculation of local point concentrations.

Consider a two-reactant system, based on reactants A (*e.g.* anion of weak acid) and H (proton), whose charges are *Q* and +1, respectively. The concentration of the j_th_ species, *C*_j_, is calculated in terms of these reactants; Eq. (A1)

*C*_j_ = *C*_AQ_^Eaj^ *C*_H+_^Ehj^ *β*_j_ (A1)

defined by the general equilibrium Eq. (A2)

*E*_aj_ A + *E*_hj_ H ⮀ A_Eaj_ H_Ehj_ (A2)

where *β*_j_ is the ‘cumulative’ formation constant [1] (pp. 148-157), and *E*_aj_ and *E*_hj_ are the A and H stoichiometric coefficients, respectively, of the j^th^ species. The core idea here is to place only the ‘bare’ reactants on the left side of the equilibrium expression and the product species formed on the right side.

The *β*(*I*_ref_) reference set of cumulative constants (*i.e.* those refined by regression analysis) are transformed to the local set *β*(*I*) according to the general Eq. (A3)

$\log\beta_{\text{j}}\left(I\right)= \log\beta_{\text{j}}\left(I_{\text{ref}}\right)+E_{\text{aj}}\log\left[\frac{f_{\text{A}}\left(I\right)}{f_{\text{A}}\left(I_{\text{ref}}\right)}\right]+E_{\text{hj}}\text{l}\mathrm{og}\left[\frac{f_{\text{H}}\left(I\right)}{f_{\text{H}}\left(I_{\text{ref}}\right)}\right]-\log\left[\frac{f_{\text{j}}\left(I\right)}{f_{\text{j}}\left(I_{\text{ref}}\right)}\right]$ (A3)

The ionic-strength-dependent activity coefficients of A, H, and j^th^ species are denoted *f*_A_, *f*_H_, and *f*_j_, respect­tively. The activity coefficients are cast in an expanded equation based on the hydration theory proposed by Robinson and Stokes [2,3], further elaborated by Bates *et al.* [4] and Robinson and Bates [5] to include single-ion activities, then slightly modified by Bockris and Reddy [6] and recently applied to solubility data of a druglike molecule by Wang *et al.,* Eq. (A4) [7]:

$\log f_{\text{i}}(I)=-Q_{\text{i}}^{2} \frac{A \sqrt{I}}{1+Bå_{\text{i}}\sqrt{I}}-h_{\text{i}} \log a_{\text{H}_{2}\text{O}}+\log\left(\frac{C_{\text{H}_{2}\text{O}} + \sum_{\text{j}} C_{\text{j}}}{C_{\text{H}_{2}\text{O}} + \sum_{\text{j}} (1-h_{\text{j}})C_{\text{j}} }\right)$ (A4)

The first term on the right side of Eq. (A4) is the Debye-Hückel equation accounting for the long-range ion-ion electrostatic interactions. At 25 °C and *I* = 0.0, 0.15, and 1.0 M (NaCl), the respective parameters (molar scale) are: *A* = 1.825×10^6^ (*εT*)^-3/2^ = 0.512, 0.528, 0.642; ion size scaler, *B* = 50.29 (*εT*)^-1/2^ = 0.329, 0.333, 0.355; dielectric constant of water, *ε* = 78.3, 76.8, 67.3. *T* / K is the absolute temperature. The mean diameter of the i^th^ hydrated ion is *å*_i_ [8].

The second and third terms are ascribed to short-range ion-solvent interactions. The second term is related to the decrease in the activity of water due to the work done in immobilizing some of the bulk water to hydrate ions. The activity of water, *a*_H2O_ = 1.000, 0.995, 0.967 [6] at the above three ionic strength values. The last term in Eq. A4 is related to free energy change of the ions, as their concentrations effectively increase when the ‘available’ volume of bulk water decreases upon hydration of the ions. The summation symbols in Eq. (A4) are over all charged species (including the reactants, inert salt and buffer components) under consideration. The concentration of pure water is *C*_H2O_ = 55.51 M. The *h*_j_ in the summation term is hydration number of the j^th^ ion [3]. Values of *h*_j_ were selected/estimated as described by Wang *et al.* [7]. For neutral molecules, it is often assumed that *h*_j_ = 0.

The activity coefficients of uncharged species can be determined by the second two terms in Eq. (A4). In practice, the activity coefficients are barely different from 1. As a modification to Eq. A4, in *p*DISOL-X, the activity of uncharged species (*e.g.* HA...H_6_A, B, XH) includes the contribution of the salting-out factor (Setschenow constant [9]), *K*_salt_ / M^-1^ , the values of which were estimated empirically using Eq. (A5b) [10].

log *f*_s_ = *K*_salt_(*I* - *I*_ref_) (A5a)

*K*_salt_ = 0.090 - 0.073*A* - 0.064*B* - 0.039*S*_π_ - 0.002*E* + 0.188*V* (A5b)

*A*, *B*, *S*_π_, *E* and *V* are the five Abraham solvation descriptors: H-bond acidity, H-bond basicity, dipolarity-pola­rizability, excess molar refractivity, and McGowan molar volume, respectively [11,12]. In the calculation of acti­vity coefficients in *p*DISOL-X, all charged species are treated with the legacy SRHT Eq. (A4). However, if a neutral solute is considered, then Eq. (A5a) is used to calculate its activity coefficient. All solids are assigned log *f*_s_ = 1.

Appendix B. pH electrode calibration, standardization and automatic compensation

The ‘blank’ acid-base titration method can be used, based on an empirical four parameter equation, in what has been called the ‘standardization’ step [13], Eq (A6).

pH = α + *k*_S_ p_c_H + *j*_H_*C*_H+_+ *j*_OH_*K*_w_/*C*_H+_ (A6)

where pH is the ‘operational’ pH (meter reading) and *K*_w_ is the ionization constant of water, which is a function of temperature and ionic strength [14]. The *j*_H_ term corrects pH readings for the nonlinear pH response due to liquid junction and asymmetry potentials in highly acidic solutions (pH <2), while the *j*_OH_ term corrects for high-pH nonlinear effects [1] (pp. 140-145). Typical values of the adjustable parameters at 25 °C, based on titration of ‘blank’ aqueous solutions containing 0.15 M KCl, are *α* = 0.09, *k*_S_= 1.002, *j*_H_ = +0.5 and *j*_OH_ = -0.5. However, each electrode possesses its own characteristic set, which can shift with repeated exposure to saturated solutions.

In solubility-pH profiles, ionic strength may reach values exceeding 10 M. The experimentally determined parameters in Eq. (A6) are automatically compensated in the data analysis program, for changes in *I* at the local pH from the benchmark level of *I*_ref_ = 0.15 M, according to empirically determined relationships [1] (pp. 64-65), Eqs. (7a) to (7d):

$\alpha=0.059+0.085\frac{\sqrt{I}}{(1+\sqrt{I})}$ (A7a)

$k_{S} =0.995+0.032\left(\frac{\sqrt{I}}{1+\sqrt{I}}-0.57I\right)$ (A7b)

$j_{\text{H}} = 0.80 \text{–} 2.0 I$ (A7c)

$j_{\text{OH}} = -0.59 +0.6 I$ (A7d)

Appendix C. Explicit solubility-pH equations in saturated solution

All the equations derived below refer to aqueous solutions saturated with a neutral weak acid (HA), a weak base (B), or an ampholyte (XH). To keep the equations simple and relatively transparent, in the following sample derivations of explicit equations, activity and salting-out corrections are not applied in the derivation of the saturation endpoint pH_w_. All total reactant concentrations are taken to be less than the salt solubility, so only the neutral species can precipitate as excess solids. The concentrations of the neutral species are frequently denoted as *C*_HA s_, C_B s_ and *C*_XH s_, to emphasize that these species are saturated in the solutions. The subscripts ‘s’ will not be used here, since the defined context here is unambiguous. The lowest possible solubility value of the neutral species is called the ‘intrinsic solubility’, denoted by the symbol *S*_0_.

Saturated solution of monoprotic weak acid, HA, in pure water

The equilibrium ‘model’ refers to a set of equilibrium equations and the associated equilibrium constants. In the case of a monoprotic weak acid, a saturated solution can be defined by the two equations and the corresponding constants

HA ⮀ H^+^ + A^-^ *K*_a_ = *C*_H+_ *C*_A-_ / *C*_HA_ (A8a)

HA(s) ⮀ HA *S*_0_ = *C*_HA_ (A8b)

*K*_a_ is the ionization constants of the weak acid and *S*_0_ is the intrinsic solubility of the acid. The solubility, *S*, at a particular pH is defined as the mass balance sum of the concentrations of all the A-containing components dissolved in the aqueous phase, *A*_tot,aq_, Eq. (A9)

*S* = *C*_HA_+*C*_A-_ = *C*_HA_ + *K*_a_ *C*_HA_/ *C*_H+_ = *S*_0_ (1 + *K*_a_ / *C*_H+_) (A9)

The square brackets denote molar concentration of species. In logarithmic form, Eq. (A10)

log *S* = log *S*_0_ + log (1 + 10^- p^*^K^*^a + pH^) (A10)

Eq. A10 is called the Henderson-Hasselbalch (HH) equation for a monoprotic weak acid. The plot of log *S* *vs.* pH is defined by a hyperbolic curve. In the plot, the limiting slope in acidic solutions (pH << p*K*_a_) is zero and in alkaline solutions (pH >> p*K*_a_) is +1. p*K*_a_ is indicated by the pH where the slope is +½.

To calculate the equilibrium pH_w_, it is useful to start with the total hydrogen excess in aqueous solution, *H_t_*_ot,aq_, expressed in terms of the equilibrium model (Eq. (A8)) and set equal to the analytical acid-base concentrations (*C*_HCl_, C_NaOH_ and *A*_tot,aq_):

H_tot,aq_ = *C*_H+_ - *C*_OH-_ + *C*_AH_ = *C*_H+_ - *K*_w_/*C*_H+_ + *S*_0_ = *C*_HCl_ - *C*_NaOH_ + *A*_tot,aq_ = C_HCl_ - *C*_NaOH_ + *S*_0_ + *C*_A-_ (A11)

On rearranging the above, noting that at endpoint pH_w_, *C*_HCl_ = *C*_NaOH_, EQs. (12)

*C*_H+_ - *K*_w_/*C*_H+_ + *S*_0_ = *C*_HCl_ - *C*_NaOH_ + *S*_0_ + *C*_A-_ (A12a)

*C*_H+_ - *K*_w_/*C*_H+_ - *C*_A-_ = 0 (A12b)

*C*_H+_ - *K*_w_/*C*_H+_ - *S*_0_*K*_a_ / *C*_H+_ = 0 (A12c)

*C*_H+_^2^ = *K*_w_ + *S*_0_*K*_a_ (A12d)

The equilibrium pH of a saturated solution of a monoprotic weak acid is

(*C*_H+w_)^ACID^ = (*K*_w_ + *S*_0_ *K*_a_)^1/2^ (A13)

Saturated solution of diprotic weak acid, H_2_A, in pure water

The above procedure can be extended to the case of a diprotic acid, although *C*_H+w_ is no longer easily stated in explicit form. As such, the equation for the total hydrogen excess in aqueous solution is given by Eq. (A14)

*H*_tot,aq_ = *C*_H+_ - *C*_OH-_ + *C*_AH-_ + 2 *C*_H2A_ = *C*_HCl_ - *C*_NaOH_ + 2*A*_tot_,_aq_ (A14)

Eq. (A14) is then transformed by inserting the expressions for p*K*_a1_, p*K*_a2_ and *S*_0_. At the saturation endpoint, *C*_HCl_ = *C*_NaOH_. The transformed equation is further re-arranged to the cubic formula in terms of *C*_H+w_.

(*C*_H+w_)^3^ - *C*_H+w_ (*K*_w_ + *K*_a1_*S*_0_) - 2 *K*_a1_*K*_a2_ *S*_0_ = 0 (A15)

The equation may be readily solved using a spreadsheet method, as described elsewhere [15]. For triprotic and more complicated multiprotic weak acids, the derived higher-order polynomial equations analogous to the above expression become unwieldy. Their exact forms appear not to have been published. Rather than dealing with explicit equations (*e.g.* Eqs. (A13), (A15), (A19), (A20), (A25)), the *p*DISOL-X program automa­ti­cally derives implicit equivalents, and readily adapts them to activity corrections.

Saturated solution of monoprotic weak base, B, in pure water

In the case of a monoprotic weak base, a saturated solution can be defined by Eqs. (16)

BH^+^ ⮀ H^+^ + B *K*_a_ = *C*_H+_*C*_B_ / *C*_BH+_ (A16a)

B(s) ⮀ B *S*_0_ = *C*_B_ (A16b)

The total solubility, *S*, at a particular pH is defined by Eq. (A17)

*S* = *C*_B_ + *C*_BH+_ (A17)

The above equation can be further transformed to Eq. (A18) as noted above.

log *S* = log {*C*_B_ + *C*_B_*C*_H+_ / *K*_a_ } = log *C*_B_ + log (1 + *C*_H+_ / *K*_a_) = log *S*_0_ + log (1 + 10^+p^*^K^*^a - pH^) (A18)

The above Henderson-Hasselbalch (HH) equation (A18) for a monoprotic weak acid describes a hyperbolic log *S* - pH curve, vertically mirroring that of the weak acid case. The limiting slope in acidic solutions (pH << p*K*_a_) is -1 and in alkaline solutions (pH >> p*K*_a_) is zero. The p*K*_a_ is indicated by the pH where the slope is -½.

Following similar steps noted for the case of a monoprotic weak acid, the corresponding explicit Eq. (A19) for the equilibrium pH can be derived for a monoprotic weak base as

(*C*_H+w_)^BASE^ = {*K*_w_ / (1 + *S*_0_ /*K*_a_)}^1/2^ (A19)

For a diprotic weak base, the corresponding explicit formula derives as the cubic Eq. (A20)

(*C*_H+w_)^3^ - (*C*_H+w_)^2^*K*_a1_(1 + *K*_a2_ / *S*_0_) / 2 - *K*_w_ *K*_a1_ *K*_a2_ / *S*_0_ = 0 (A20)

As noted above, the equation may be readily solved using a spreadsheet method [15].

Saturated solution of diprotic ampholyte, XH, in pure water

A saturated ampholyte solution can be defined by the equations (A21) and the corresponding constants:

XH_2_^+^ ⮀ H^+^ + XH *K*_a1_ = *C*_H+_ *C*_XH_ / *C*_XH2+_ (A21a)

XH ⮀ H^+^ + X^-^ *K*_a2_ = *C*_H+_ *C*_X-_ / *C*_XH_ (A21b)

HX(s) ⮀ XH *S*_0_ = *C*_XH_ (A21c)

The total solubility, *S*, at a particular pH is defined as the mass balance sum of the concentrations of all the species dissolved in the aqueous phase:

*S* = *C*_X-_ + *C*_XH_ + *C*_XH2+_ = *X*_tot,aq_ (A22)

The above equation can be transformed into an expression containing only constants and *C*_H+_ (as the only variable), by substituting the above ionization and solubility Eq. (A23).

log *S* = log {K_a2_ C_XH_/C_H+_ + *C*_XH_+ *C*_XH_*C*_H+_ / *K*_a1_} = log *C*_XH_ + log (*K*_a2_ / *C*_H+_ + 1 + *C*_H+_ / *K*_a1_) =
 = log *S*_0_ + log (1 + 10^-p^*^K^*^a2+pH^ + 10^+p^*^K^*^a1-pH^) (A23)

The above HH equation for an ampholyte describes a U-shaped log *S*-pH curve. The limiting slope in acidic solutions is -1 and in alkaline solutions is +1. The limiting slope between the two p*K*_a_ values is zero. p*K*_a1_ is indicated by the pH where the slope is -½; p*K*_a2_ is indicated by the pH at slope +½.

As before, pH_w_ can be calculated from the total hydrogen excess, *H*_tot,aq_, expressed in terms of the equilibrium model (Eqs. (A21)) and set equal to the analytical acid-base concentrations (*C*_HCl_, *C*_NaOH_ and *X*_tot,aq_), Eq. (A24):

*H*_tot,aq_ = *C*_H+_ - *C*_OH-_ + C_XH_ + 2 *C*_XH2+_ = *C*_H+_ - *K*_w_/*C*_H+_ + *S*_0_ + 2*S*_0_*C*_H+_ / *K*_a1_ = *C*_HCl_ - *C*_NaOH_ + *X*_tot,aq_ (A24)

On rearranging the above (noting that at endpoint pH_w_, C_HCl_ = *C*_NaOH_), the equilibrium pH of a saturated solution of a monoprotic weak acid is given by Eq. (A25)

(*C*_H+w_)^AB^ = {(*K*_w_ + *S*_0_ *K*_a2_) / (1 + *S*_0_/*K*_a1_)}^1/2^ (A25)

References

1. A. Avdeef. *Absorption and Drug Development, 2^nd^ Ed*., Wiley-Interscience, Hoboken NJ, 2012. ISBN 978-1-118-05745-2. <https://doi.org/10.1002/9781118286067>
2. R.H. Stokes, R.A. Robinson. *Journal of the American Chemical Society* **70** (1948**)** 1870-1878. <https://doi.org/10.1021/ja01185a065>
3. R.A. Robinson, R.H. Stokes. *Electrolytic Solutions*. 2^nd^ Rev. Ed. Dover Publications, Inc., Mineola, NY. 2002, pp. 18-19. <https://easypdfs.cloud/downloads/4879156-electrolyte-solutions-robinson-stokes>
4. R.G. Bates, B.R. Staples, R.A. Robinson. Ionic hydration and single ion activities in unassociated chlorides at high ionic strengths. *Analytical Chemistry* **42** (1970) 867-871. <https://doi.org/10.1021/ac60290a006>
5. R.A. Robinson, R.G. Bates. Ionic activity coefficients in aqueous mixtures of NaCl and MgCl_2_. *Marine Chemistry* **6** (1978) 327-333. <https://doi.org/10.1016/0304-4203(78)90013-0>
6. J.O’.M. Bockris, A.K.N. Reddy. *Modern Electrochemistry*, Vol. 1. Plenum Publishing Corp., New York, NY, 1973. <https://doi.org/10.1007/b114546>
7. Z. Wang, L.S. Burrell, W.J. Lambert. Solubility of E2050 at various pH: a case in which apparent solubility is affected by the amount of excess solid*. Journal of Pharmaceutical Sciences* **91** (2002) 1445-1455. <https://doi.org/10.1002/jps.10107>
8. J. Kielland. Individual activity coefficients of ions in aqueous solutions. *Journal of the American Chemical Society* **59** (1937) 1675-1678. <https://doi.org/10.1021/ja01288a032>.
9. N. Ni, S.H. Yalkowsky. Prediction of Setschenow constants. *International Journal of Pharmaceutics* **254** (2003) 167-172. <https://doi.org/10.1016/S0378-5173(03)00008-5>
10. A. Avdeef. Anomalous salting-out, self-association and pK_a_ effects in the practically-insoluble bromothymol blue. *ADMET & DMPK* **11** (2023) 419-432. <https://doi.org/10.5599/admet.1822>
11. M.H. Abraham, J. Le. The correlation and prediction of the solubility of compounds in water using an amended solvation energy relationship. *Journal of Pharmaceutical Sciences* **88** (1999) 868-880. <https://doi.org/10.1021/js9901007>
12. J.A. Platts, D. Butina, M.H. Abraham, A. Hersey. Estimation of molecular linear free energy relation descriptors using a group contribution approach. *Journal of Chemical Information and Computer Sciences* **39** (1999) 835-845. <https://doi.org/10.1021/ci980339t>
13. A. Avdeef, J.J. Bucher. Accurate measurements of the concentration of hydrogen ions with a glass electrode: calibrations using the Prideaux and other universal buffer solutions and a computer-controlled automatic titrator. *Analytical Chemistry* **50** (1978) 2137-2142. <https://doi.org/10.1021/ac50036a045>
14. F.H. Sweeton, R.E. Mesmer, C.F. Baes, Jr. Acidity measurements at elevated temperatures. VII. Dissociation of water. *Journal of Solution Chemistry* **3** (1974) 191-214. <https://doi.org/10.1007/BF00645633>.
15. A. Avdeef, K. Sugano. Salt solubility and disproportionation - uses and limitations of equations for pHmax and the in-silico prediction of pHmax. *Journal of Pharmaceutical Sciences* **111** (2022) 225-246. <https://doi.org/10.1016/j.xphs.2021.11.017>
